# 

**Published:** 1888-03

**Authors:** 


CONTENTS.

VOL. 35 No. 3.

| | PAGE. |
|---|---|
| Special | 50 |
| What's in a Name | 50 |
| How Ought we to Dispose of the Dead? | 53 |
| Our Dear Boy | 56 |
| Came True | 57 |
| A Freak of Nature | 57 |
| Imitation Precious Stones | 58 |
| Snakes | 58 |
| Female Beauty | 59 |
| In Petticoats | 60 |
| Found Through Two Dreams | 61 |
| A Queer Incident | 62 |
| Cause and Cure of Disease | 63 |
| A Sentimental Sketch | 67 |
| Book Notices | 67 |
| Household | 69 |
| Miscellaneous | 71 |

INDEX TO ADVERTISEMENTS OF HALF A PAGE AND OVER.

| | PAGE. |
|---|---|
| Crystal Pepsin Tablets | I |
| Blinded by Prejudice | II |
| The Tea Cure | III |
| Lactopeptine | V |
| Annexation of Canada | VI |
| Dr. Molesworth | VII |
| Bovinine | VIII |
| Stevensville Mills | IX |
| Hollow Suppositories | X |
| Celerina | XI |

